# First insights into the genetic characteristics and drug resistance of *Mycobacterium tuberculosis* population collected during the first national tuberculosis prevalence survey of Lao PDR (2010–2011)

**DOI:** 10.1186/s12879-019-4435-z

**Published:** 2019-10-15

**Authors:** Silaphet Somphavong, Jean-Luc Berland, Marie Gauthier, Thi Thuong Vu, Quang Huy Nguyen, Vibol Iem, Phouvang Vongvichit, Donekham Inthavong, Vanthala Akkhavong, Phetsavanh Chanthavilay, Sengaloun Soundala, Inthalaphone Keovichit, Glaucia Paranhos-Baccalà, Phimpha Paboriboune, Thi Van Anh Nguyen, Anne-Laure Bañuls

**Affiliations:** 1 Centre d’Infectiologie Lao-Christophe Mérieux, Vientiane, Lao PDR; 2 MIVEGEC (IRD-CNRS-Université de Montpellier), Centre IRD, Montpellier, France; 3 LMI “Drug Resistance in South East Asia, DRISA”, Hanoi, Vietnam; 4 Laboratoire des Pathogènes Émergents, Fondation Mérieux, Lyon, France; 5 Department of Bacteriology, National Institute of Hygiene and Epidemiology, Hanoi, Vietnam; 6 Department of Pharmacological, Medical and Agronomical Biotechnology, University of Science and Technology of Hanoi, Academy of Science and Technology, Hanoi, Vietnam; 7 National reference laboratory for tuberculosis, Vientiane, Lao PDR; 8 National Tuberculosis Control Program, Vientiane, Lao PDR; 9 Faculty of postgraduate studies, University of Health Sciences, Vientiane, Lao PDR; 10 Biomérieux, Rio de Janeiro, Brasil

**Keywords:** Molecular epidemiology, *Mycobacterium tuberculosis* family, Drug-resistant tuberculosis, Lao PDR

## Abstract

**Background:**

In Lao People’s Democratic Republic (PDR), tuberculosis (TB) prevalence was estimated at 540/100,000 in 2011. Nevertheless, little is known about the genetic characteristics and anti-TB drug resistance of the *Mycobacterium tuberculosis* population*.* The main objective of this work was to study the genetic characteristics and drug resistance of *M. tuberculosis* population collected during the first National TB Prevalence Survey (TBPS) of Lao PDR (2010–2011).

**Methods:**

Two hundred and twenty two isolates collected during TBPS (2010–2011) were analyzed with the GenoType MTBDR*plus* test for *M. tuberculosis* identification and drug resistance detection. Then, 206 of the 222 isolates were characterized by spoligotyping and MIRU-VNTR typing.

**Results:**

Among the 222 *M. tuberculosis* isolates, 11 were mono-resistant to isoniazid and 2 were resistant to isoniazid and rifampicin (MDR-TB), using the GenoType MTBDR*plus* test. Among the 202 genetically characterized isolates, the East African-Indian (EAI) family was predominant (76.7%) followed by the Beijing (14.4%) and T (5.5%) families. EAI isolates came from all the country provinces, whereas Beijing isolates were found mainly in the northern and central provinces. A higher proportion of Beijing isolates was observed in people younger than 35 years compared to EAI. Moreover, the percentage of drug resistance was higher among Beijing (17.2%) than EAI (5.2%) isolates, and the two MDR-TB isolates belonged to the Beijing family. Combined analysis of the MIRU-VNTR and spoligotyping results (*n* = 202 isolates) revealed an estimated clustering rate of 11% and the occurrence of mini-outbreaks of drug-resistant TB caused by Beijing genotypes.

**Conclusions:**

The EAI family, the ancient and endemic family in Asia, is predominant in Lao PDR whereas the prevalence of Beijing, the most harmful *M. tuberculosis* family for humans, is still low, differently from neighboring countries. However, its association with drug resistance, its presence in young patients and its potential association with recent transmission suggest that the Beijing family could change TB epidemiological pattern in Lao PDR. Therefore, efficient TB control and surveillance systems must be maintained and reinforced to prevent the emergence of highly transmissible and drug-resistant strains in Lao PDR, as observed in neighboring countries.

**Electronic supplementary material:**

The online version of this article (10.1186/s12879-019-4435-z) contains supplementary material, which is available to authorized users.

## Background

Tuberculosis (TB) remains a major public health problem. Although the number of TB deaths fell by 22% between 2000 and 2015, TB still was one of the top 10 causes of death worldwide in 2015 [1], with an estimated 10.4 million of new TB cases worldwide. Six countries account for 60% of all new cases (India, Indonesia, China, Nigeria, Pakistan and South Africa) and more than half of these cases were in Asia. The emergence of drug-resistance is a global issue for TB control. In 2015, the number of new cases of multidrug-resistant TB (MDR-TB) was estimated at 480000, with an additional 100,000 people with rifampicin (RIF)-resistant TB who are eligible for MDR-TB treatment. India, China and the Russian Federation accounted for 45% of all these cases. In Southeast Asian countries, TB is one of the top ten communicable diseases with an increasing emergence of MDR-TB and extensively drug-resistant TB (XDR-TB) [2].

Lao People’s democratic Republic (PDR) (population of 6.8 million in 2015) is not among the high TB burden countries. However, this landlocked country is surrounded by China, Myanmar, Cambodia, Vietnam and Thailand that are among the 30 high TB burden countries in the world. The National Tuberculosis Control Program (NTCP) of Lao PDR started the Directly Observed Treatment Short course (DOTS) strategy in 1995 with the support of the Damien Foundation Belgium (DFB) and the World Health Organization (WHO). After the first national TB prevalence survey (2010–2011), WHO re-estimated the prevalence of all TB forms at 540/100,000, 1.9 times higher than previous estimates [3]. Moreover, little is known about anti-TB drug resistance. The only available cross-sectional study (*n* = 87 TB isolates) conducted in three hospitals in 2010 showed that 8% of isolates were resistant to isoniazid (INH) and 1.2% caused XDR-TB [4]. Similarly, the *M. tuberculosis* population in Lao PDR is still unknown. Many studies have reported that the Beijing and East African-Indian (EAI) families are predominant in Asian countries [5–10]. In Vietnam, the Beijing family is currently invading the country and is more likely to be drug resistant than the EAI family [10, 11]. In this context, the main objective was to study the genetic characteristics and drug resistance of *M. tuberculosis* population collected during the first National TB Prevalence Survey of Lao PDR (2010–2011) in order to better understand the *M. tuberculosis* population structure and the TB epidemiology in this country. The specific objectives were: a) to characterize using different molecular methods *M. tuberculosis* isolates collected during the first national survey; b) to describe the spatial distribution of families and drug resistance; c) to explore the link between genetic diversity and demographical data; d) to estimate the clustering rate according to the *M. tuberculosis* families and drug resistant patterns.

## Methods

### Study population

**Fig. 1:**
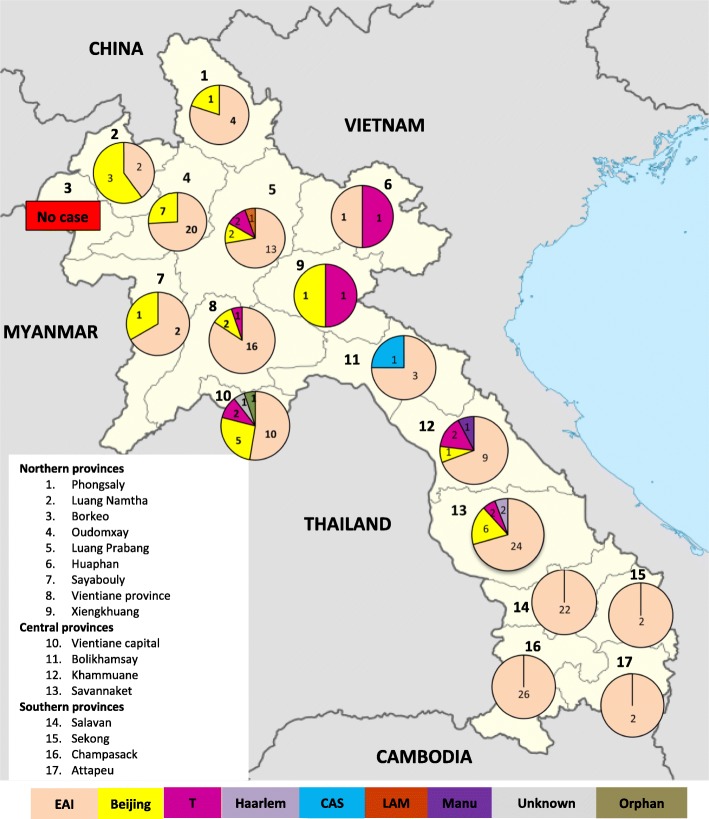
Distribution of *M. tuberculosis* families in the different provinces of Lao PDR (PDF). The numbers on the map (1 to 17) correspond to the provinces divided in three regions (North, Center, and South). The numbers in the pie charts indicate the number of isolates found in each province. Each *M. tuberculosis* family is represented by a different color (see color code in figure)

The TB isolates used in this study were collected during the first national TB prevalence survey in Lao PDR (July 2010–December 2011). The survey design and sample size were determined according to the WHO recommendations and has been described in Law et al. (2015). During this survey that covered the 17 provinces of Lao PDR (organized in three main regions, North (1–9), Center (10–13) and South (14–17), see Fig. 1), at least one sputum specimen was collected from 6290 (99.1%) of the 6346 participants suspected to have TB on the basis of clinical data (chronic cough and/or hemoptysis and/or chest X-ray abnormalities). Finally, TB was confirmed in 237 participants, according to the study case definition [3]. Among these 237 patients, 94 had at least one smear-positive sputum and culture-confirmed *M. tuberculosis* (definite cases), 13 had at least one smear-positive sputum and chest X-ray (CXR) findings suggestive of TB with negative culture (probable cases), and 130 had smear-negative but culture-positive specimens*.* In summary, the presence of *M. tuberculosis* was confirmed by culture in 224 isolates and 222 isolates of these isolates (corresponding to 222 different patients) could be included in this study. The collected sputum specimens were decontaminated with 4% sodium hydroxide and then they were inoculated on two slopes of solid Kudoh-modified Ogawa medium without centrifugation [12]. All subcultures were sent by the National Tuberculosis Reference Laboratory (NRL) to the Center of Infectiology Lao-Christophe Mérieux (CILM) for species identification and genetic characterization. Colonies were scraped from the medium slopes and resuspended in 300 μL of distilled water, heated at 95 °C for 20 min, and centrifuged at 13000 g for 5 min. Then, the DNA-containing supernatant was transferred into a new tube and stored at − 80 °C. The patients’ demographic, epidemiologic, and clinical data were collected using a questionnaire, including residence, sex, age, TB history, TB symptoms, and CXR findings.

### *Mycobacterium tuberculosis complex* identification and drug resistance testing

The GenoType® MTBDR*plus* test (Hain Lifescience GmbH), a DNA STRIP®-based technology, was used according to the manufacturer’s instructions to identify the *M. tuberculosis* complex and resistance to RIF and/or INH [13].

### Spacer oligonucleotide typing (spoligotyping)

Spoligotyping (the classical 43-spacer format) was performed as previously described [14, 15]. DNA samples of the *M. tuberculosis* H37Rv and *Mycobacterium bovis* BCG strains were included as positive controls. Molecular biology-grade water was used as a negative control. The spoligotypes were then recorded in 43-digit binary format and compared with those recorded in the SpolDB4 database (http://www.pasteur-guadeloupe.fr:8081/ SITVIT_ONLINE/) to identify the Spoligotype International Type (SIT) and family [16]. For the spoligotypes that matched the SITs, but could not be related to any family (i.e., unknown), and for the spoligotypes that were not present in the SpolDB4 database (i.e., orphan), the SPOTCLUST program, which was built from the spolDB3 database (http://tbinsight.cs.rpi.edu/run_spotclust.html) [17], was used to search for *M. tuberculosis* family similarity. In the SPOTCLUST analyses, the family assignation was retained when the probability was ≥90%. Nevertheless, the final designation of families and subfamilies was also based on the MIRU-VNTR data (see below).

### Mycobacterial interspersed repetitive unit-variable number tandem repeat (MIRU-VNTR) typing

MIRU-VNTR typing was performed as previously described [18, 19] and the full set of 24 MIRU-VNTR loci was used for isolate characterization. The patterns obtained for the 24 loci were used to create a 24-digit allelic profile for each isolate. The MIRU-VNTR typing results were analyzed using MIRU-VNTRplus (http://www.miru-vntrplus.org), a freely accessible web-based program [20]. A Neighbor-Joining (NJ) tree based on categorical distances was built by combining the spoligotyping and MIRU-VNTR results.

The final designation of family/subfamily was revised using the MIRU-VNTRplus website (MIRU-VNTRplus.org) based on the family results for each isolate and the MIRU-VNTR/spoligotyping phylogenetic tree.

### Data analysis

A cluster was defined as two or more isolates with identical genotype by spoligotyping and MIRU-VNTR typing. Recent transmission was estimated by calculating the clustering rate as follows: CR = (nc-c)/n, where CR is the clustering rate, nc is the total number of clustered isolates, c is the total number of clusters, and n is the total number of isolates [21]. The patients’ age was shown as median and interquartile range (IQR). Associations between *M. tuberculosis* families, patient data and overall drug resistance status were assessed using the Chi-square or Fisher’s exact test; when the sample size was lower than 5. The statistical analysis was not performed for RIF and INH resistance independently due to the small number of resistant isolates. A *P*-value < 0.05 was considered statistically significant. Statistical analyses were done using Stata (v12.1, Stata Corporation, USA).

## Results

### *M. tuberculosis* complex identification and epidemiological data

The GenoType® MTBDR*plus* test allowed confirming that the 222 isolates included in the study belonged to the *M. tuberculosis* complex. The patients’ median age was 56 years (IQR: 40–68), with a men to women ratio of 2:1. Patients were mainly from rural areas (83.3% vs 16.7% from urban areas), and the number of *M. tuberculosis* isolates across the 17 provinces varied from 0 to 34 (mean number = 13) (Fig. 1 and Additional file 2: Table S1).

### Characterization of anti-TB drug resistance (GenoType® MTBDR*plus* test)

Analysis of the RIF and INH resistance profile of 222 isolates with the GenoType® MTBDR*plus* test showed that 209 isolates (94.1%) were sensitive to both drugs, 11 (5%) were resistant only to INH, and 2 (0.9%) were resistant to both INH and RIF (MDR-TB). Among the 13 INH-resistant isolates, 10 (76.9%) had mutations in the *kat*G gene (S315 T in all isolates) and 3 (23.1%) had mutations in *inh*A promoter region (C15T in two isolates and T8C in one). The two RIF-resistant isolates carried the D516V mutation in *rpo*B gene.

### Identification of the *M. tuberculosis* families/subfamilies

Spoligotyping and 24-locus MIRU-VNTR typing were performed on 206 of the 222 isolates (Table 1, Additional file 1: Figure S1). The *M. tuberculosis* family/subfamily identifications were determined using SITVITWEB (SpolDB4 database), SPOTCLUST and MIRU-VNTRplus. The patterns of four isolates reflected either clonal variants (with double alleles at a single MIRU-VNTR locus) or mixed infections (with double alleles at two MIRU-VNTR loci) [22] (Additional file 2: Table S1). These four isolates were removed from the analysis to avoid incorrect designation. The other 202 isolates had 58 different spoligotype profiles among which 41 spoligotypes were unique and 17 patterns allowed the clustering of 161 isolates. Each cluster contained 2 to 40 isolates (average = 9). Moreover, 165 isolates (81.68% of 202) were assigned to 29 SITs and seven families present in the SpoIDB4 database; two (1.0%) were unknown; and 35 (17.3%) were orphans. The 35 orphan and the two unknown isolates were then compared using SPOTCLUST. Finally, isolates could be classified in seven *M. tuberculosis* families and ten subfamilies (Table 1). EAI was the predominant family (76.7%, *n* = 155 isolates), followed by Beijing (14.4%, *n* = 29) and T (5.5%, *n* = 11). Five isolates (2.5%) belonged to other families, such as Haarlem (H), Central Asian Strain (CAS), Latin American-Mediterranean (LAM), and Manu. Only one orphan and one unknown isolate could not be identified. Within the EAI family, the most frequent subfamily was EAI5 (53.0%, *n* = 107), followed by EAI1-SOM (8.9%, *n* = 18) and EAI2-Nonthaburi (6.4%, *n* = 13) (Table 1). The subfamily EAI4-VNM, which is found specifically in Vietnam, was poorly represented in our sampled strains (4.5%, *n* = 9) (Table 1). In the southern provinces (N. 14–17) where only the EAI family was represented (Fig. 1), the EAI5 subfamily was the most common (65.4%, *n* = 34), followed by EAI1-SOM (19.2%, *n* = 10), whereas EAI4-VNM was absent. Unlike the EAI family, which was present in all regions of Lao PDR, the Beijing family was predominantly observed in the northern (58.6%, *n* = 17) and central provinces (41.4%, *n* = 12), and was absent in the southern provinces (Fig. 1).

**Table 1:**
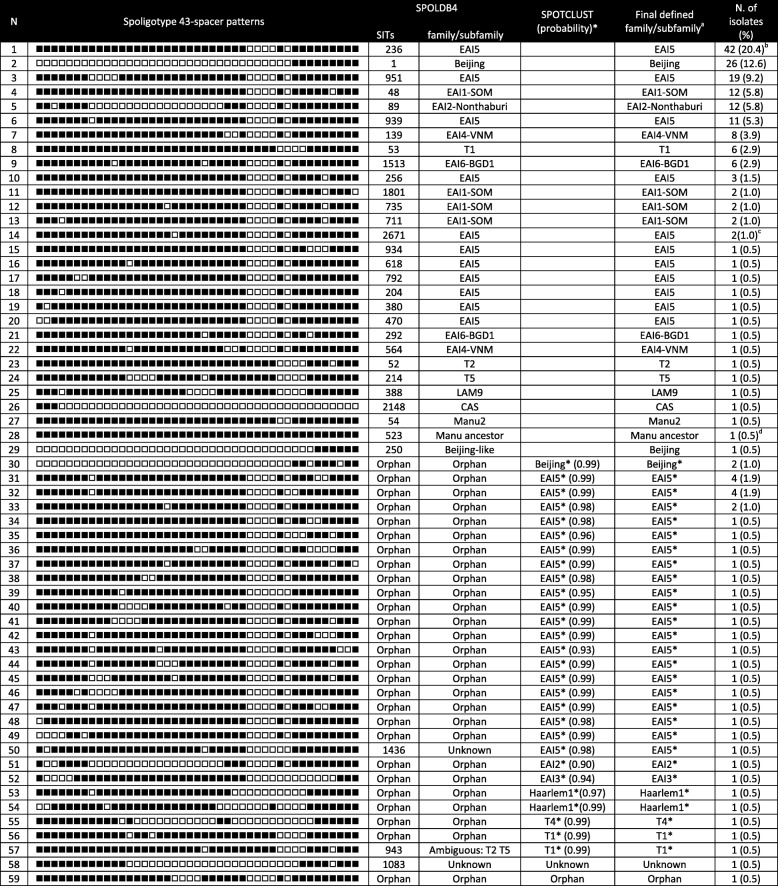
Distinct spoligotyping patterns obtained for the *206 M. tuberculosis* isolates under study

*Spoligotype defined by SPOTCLUST (probability ≥0.9)

^a^The final identification in family/subfamily was determined by combining results of SITVITWEB (SpolDB4 database), SPOTCLUST and MIRU-VNTR*plus* (see Additional file 2: Table S1)

^b^One isolate with double allele on ETRA and one isolate with double allele on QUB26 were removed from the analysis

^c^One isolate with double allele on ETRA was removed from the analysis

^d^One isolate with hybridization for all 43 spacers + double alleles on ETRA and Mtub29 was removed from the analysis

### The distribution of the *M. tuberculosis* EAI and Beijing families varies according to age, geographical origin and drug-resistance

The *M. tuberculosis* family (EAI or Beijing) distribution in the three age groups (15–34, 35–64, and ≥ 65 years of age) was significantly different (*p* = 0.002, Table 2). Specifically, the percentage of Beijing family was higher in the “15–34” group compared to EAI (34.5%, 10/29 vs 10.3%, 16/155), and the percentage of EAI family higher in the “35–64” group compared to Beijing (54.8%, 85/155 vs 34.5%, 10/29). Their geographical distribution also was significantly different (*p* = 0.001, Table 2). In the North and Center, the percentage of Beijing isolates was higher than that of EAI isolates (58.6 and 41.4% vs 37.4 and 29.0% respectively), whereas the Beijing family was not observed in the South. Similarly, drug-resistance was higher in the Beijing than EAI family (*p* = 0.03): 17.2% (5/29) of Beijing isolates were resistant to RIF and/or INH compared with 5.2% (8/155) of EAI isolates. Conversely, the proportion of Beijing and EAI isolates was not significantly different when patients were divided according to sex and strata (urban versus rural) (Table 2).

**Table 2:** Characteristics of the patients infected with EAI (76.7%) or Beijing isolates (14.4%)

Characteristics	Patients infected with EAI, *n* = 155 (%)	Patients infected with Beijing, *n* = 29 (%)	*P*-value
Age group (years)			
15–34	16 (10.3)	10 (34.5)	0.002
35–64	85 (54.8)	10 (34.5)	
≥ 65	54 (34.8)	9 (31.0)	
Sex			
Men	105 (67.7)	15 (51.7)	0.09
Women	50 (32.3)	14 (48.3)	
Strata			
Rural	134 (86.5)	22 (75.9)	0.14
Urban	21 (13.5)	7 (24.1)	
Regions			
North	58 (37.4)	17 (58.6)	< 0.001
Centre	45 (29.0)	12 (41.4)	
South	52 (33.6)	0	
Anti-TB drug resistance status^a^			
Sensitive ^b^	147 (94.8)	24 (82.8)	0.03
Resistant^c^	8 (5.2)	5^d^ (17.2)	

^a^Tested with the MTBDR *plus* test for Rifampicin (RIF) and isoniazid (INH) resistance

^b^Sensitive to INH and RIF

^c^Isolates were considered resistant when they were INH and/or RIF-resistant

^d^Contains two isolates resistant to both INH and RIF (MDR-TB)

### 24-locus MIRU-VNTR typing and cluster analysis

#### 24-locus MIRU-VNTR patterns

The 206 isolates that underwent spoligotyping were also typed by 24-locus MIRU-VNTR typing. In 182 isolates (88.4%), all 24 loci could be amplified, whereas in 24 (11.7%) at least one locus could not be amplified (repeated three times). ETRA was the most frequently non-amplified locus (9/206 isolates), followed by QUB4156 (6/206) and QUB11b (5/206). These results were treated as missing data. The four isolates with double alleles (three had double alleles at only one locus and one at two loci (Additional file 2: Table S1)) were removed from the global analysis. Thus, the analyses were performed on 202 isolates. By using the results of the 24-locus MIRU-VNTR technique alone, the 202 isolates generated 173 profiles (152 unique profiles and 21 clusters). The 21 clusters contained 50 isolates (2–4 isolates per cluster; average: 2.4). Two clusters included four isolates, four clusters contained three isolates, and 15 were composed by two isolates.

#### Phylogenetic tree and cluster analysis

**Fig. 2:**
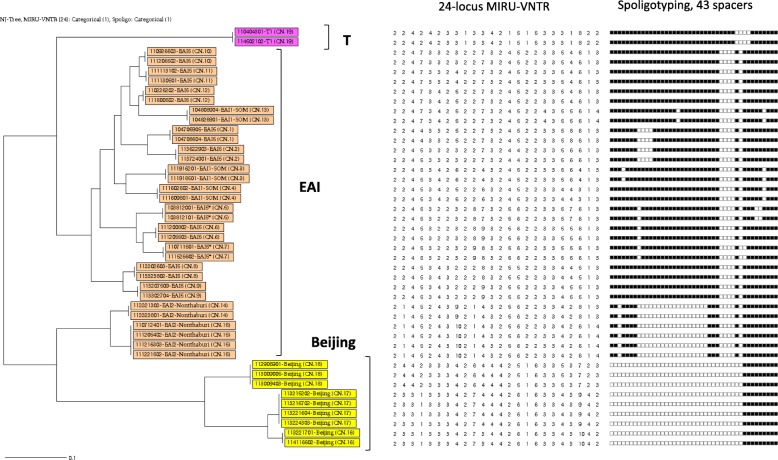
Neighbor-joining tree based on the MIRU-VNTR and spoligotyping data for 43 clustered isolates (PDF). From left to right: i) Neighbor-joining tree based on the 24-locus MIRU-VNTR and spoligotyping data for the 43 isolates grouped in 19 clusters (built using the MIRU-VNTRplus analysis tool; ii) Number of repetitions of each VNTR according to the nomenclature by Supply et al. (2006) [18]; and iii) 43-spacer spoligotypes: black spots represent the presence and white spot represent the absence of 1–43 spacers (according to the numbering by Van Embden et al. 2000) [23]. Yellow squares, Beijing clusters; orange squares, EAI clusters; dark pink, T clusters

The Neighbor-Joining (NJ) tree built by combining the MIRU-VNTR and spoligotyping data for the 202 isolates clearly differentiated the Beijing clade from the other families (Additional file 1: Figure S1). Nineteen clusters including 43 isolates (2 to 4 isolates per cluster; average: 2.3 isolates per cluster) were showed (see Fig. 2 and Additional file 2: Table S1). The EAI, Beijing and T families were present in these clusters, accounted for 32, 9 and 2 isolates respectively and were grouped in 15, 3 and 1 cluster respectively (Table 3). Thirteen out of 15 EAI clusters and all 3 Beijing clusters could be geographically linked (isolates were either from patients living in the same village or district or provinces) (see Fig. 2 and Additional file 2: Table S1). Regarding drug resistant isolates, only one cluster of Beijing family (CN.18, Fig. 2 and Additional file 2: Table S1) contained three INH-resistant isolates.

**Table 3:** Estimation of the clustering rate for the EAI, Beijing and T families

Characteristics	EAI	Beijing	T
Total number of isolates	155	29	11
Unique isolates	123	20	9
Clustered isolates	32	9	2
N. of clusters	15	3	1
Clustering rate	11.0%	20.7%	9.1%

Finally, these data allowed calculating the overall clustering rate (11.9%) and the clustering rate for the Beijing, EAI and T families (Table 3).

## Discussion

### *M. tuberculosis* families in Lao PDR

This is the first study on the genetic structure of the *M. tuberculosis* population in Lao PDR. First of all, a high proportion of orphan and unknown *M. tuberculosis* isolates (18.3%) was detected in our sample, probably because of the lack of previous genetic data. Indeed, in countries where many genetic studies have been already performed, the proportion of orphan isolates is lower, for instance 9.5% in Vietnam [10], and 8.2% in China [24]. Conversely, the proportion of isolates belonging to minor families (T, H, CAS, LAM, and MANU) was lower in Lao PDR than in Myanmar and Vietnam (7.9% vs 15 and 23%, respectively) [8, 10]. Moreover, only one isolate belonged to the CAS family, which is totally absent in Cambodia and Vietnam [9, 10]. This result is in agreement with the reported low prevalence of CAS isolates in Southeast Asia, differently from South-Central Asia (56.5% in Pakistan, 26% in India) [25, 26].

Our findings indicate that the *M. tuberculosis* population in Lao PDR is mainly composed of strains belonging to the EAI (76.7%) and Beijing (14.4%) families, similarly to neighboring countries but in different proportions. Indeed, in Cambodia and Myanmar, the EAI family is predominant (60 and 48.4% respectively), but the Beijing family also is highly prevalent (30, and 31.9%) [8, 9]. In Vietnam, the Beijing and EAI families represent 38.5%/each of the *M. tuberculosis* population (Beijing isolates were found particularly in urban areas with high population density, such as Hanoi and Ho Chi Minh) [10]. Conversely, in China, the Beijing family represents 74.1% of the *M. tuberculosis* population and was detected in all studied provinces, whereas only 0.03% of isolates belongs to the EAI family (only in Fujian province) [24]. The low proportion of Beijing isolates found in our study could be explained by the low population density (27 people per km^2^) in Lao PDR and the fact that 67% of the Lao population live in rural areas [27]. Moreover, the distribution of the *M. tuberculosis* families was heterogeneous in the different provinces of Lao PDR. EAI family isolates were from all over the country, whereas Beijing isolates came mainly from the northern and central provinces (see Fig. 1). In most of the biggest provinces (Luang Prabang, Vientiane Capital, Savannakhet), isolates belonged to different *M. tuberculosis* families, except in Champasack province where all isolates were identified as EAI (Fig. 1). Concerning the EAI subfamilies, the proportion of EAI5 was two times higher in Lao PDR (69.0%) than in Cambodia (28.8%) and in Vietnam (30.6%). On the other hand, EAI4-VNM, which was mainly identified in Vietnam (65.9%), was less frequent (4.5%) and found only in the central provinces. These data suggest that EAI5 is the most ancient *M. tuberculosis* family circulating in Lao PDR. The long history of social-economic exchange with neighboring countries has undoubtedly favored the spread of specific genotypes in the country. The “4th Population and Housing Census” (PHC) of 2015 estimated the global number of migrants at 42,000 [27]. Most of them came from Thailand (37%), Vietnam (26%), China (23%), Myanmar (6%) and Cambodia (1%). Currently, Vientiane Capital hosts the largest proportion of migrants, and this could explain the high diversity of *M. tuberculosis* families (*n* = 5) observed in this province compared with most of the other provinces (0 to 4 families) (Fig. 1). Migrants from China and Myanmar live mostly in northern provinces, those from Thailand are mainly in the central part of the country, and migrants from Vietnam are found in the center and in Attapeu province in the South [27]. The number of migrants from Cambodia (1%) is very low compared with those from other neighboring countries and they are distributed all over the country. These data could partly explain the distribution of the Beijing and EAI4-VNM subfamilies in Lao PDR and raise the question of the risk of a progressive invasion by Beijing strains, as previously observed in Vietnam [10].

### Genetic diversity and transmission of *M. tuberculosis* families in Lao PDR

To explore the genetic diversity of *M. tuberculosis* population in Lao PDR, 202 isolates were characterized by spoligotyping and MIRU-VNTR typing. The results revealed 178 genotypes, a result similar to the one reported for Cambodia (91 patterns in 105 isolates) and higher than that for Vietnam (153 genotypes for 221 isolates) [9, 10]. As expected, the EAI family was more diverse than the Beijing family (138 genotypes for 155 isolates vs 23 genotypes for 29 isolates). The 19 clusters grouped 43 isolates that belonged only to the three main families (EAI, Beijing and T). The overall clustering rate was 11.9%, reflecting a non-negligible level of recent transmission compared with high TB burden countries, such as Vietnam (16.3%) [10] and China (18.4%) [28]. Moreover, the Beijing family clustering rate was higher than the clustering rates of the other families (20.7% for Beijing vs 11.0% for EAI vs 9.1% for T), suggesting a potential association of the Beijing family in recent transmission cases, as demonstrated in many studies [10, 29–31]. Nevertheless, it is worth noting that the combination of 24 Loci MIRU-VNTR and spoligotyping can lack discrimination (only the whole genome sequencing can give us the real genotype of each isolate) making possible that some clusters include slightly different genotypes. This lack of discrimination can lead to a global overestimated clustering rate in our study. However, the large difference observed between the families (20.7% for Beijing vs 11.0% for EAI vs 9.1% for T) supports the hypothesis that Beijing, as demonstrated in many studies, might be associated with recent transmission than the other families in Laos. EAI isolate predominance, higher diversity and lower clustering rate compared with the Beijing family reinforce the hypothesis that the EAI family (specifically the EAI5 sub-family) is the more ancient *M. tuberculosis* family in Lao PDR. Most isolates in clusters (16 of the 19 clusters, and 37 of the 43 clustered isolates) were geographically linked, reflecting the occurrence of recent transmissions. Clusters were mainly observed in the northern and southern provinces, and mostly in rural area. Surprisingly, no cluster was observed in the capital city. This could be explained by the global low population density in cities and the higher patients’ recruitment in rural areas than in urban areas in our study.

### Epidemiological consideration and drug resistant TB

The proportion of the two main families was significantly different in function of the age group, region of origin and drug-resistant status. The proportion of isolates belonging to the EAI family was higher in the 35–64 age group, as observed in Cambodia, Vietnam and Myanmar, reflecting the endemic circulation of EAI in this part of the world. On the other hand, in Lao PDR the proportion of Beijing isolates in the 15–34 and 35–64 age groups was similar, whereas in Vietnam the proportion of Beijing isolates decreases with age [10].

Finally, despite the low prevalence of drug resistance in Lao PDR, the Beijing family was more represented among drug-resistant isolates, as previously reported in Cambodia, Vietnam, and China [9, 10, 32]. The Beijing isolates in clusters were geographically linked and one of the three Beijing clusters included drug-resistant isolates (see Fig. 2 and Additional file 2: Table S1). These findings underline the risk of Beijing strain expansion in Lao PDR and consequently the increasing risk of primary drug resistance in recent transmission.

## Conclusion

This study provides the first genetic insights into the *M. tuberculosis* population in Lao PDR. The presence of the main families detected in neighboring countries, particularly the EAI and Beijing families, and the 11% of recent transmission rate show that TB represents a challenge in Lao PDR. Although, the EAI family is predominant, the diversity of families observed in big cities (Vientiane, Luang Prabang, Khammuane and Savannhaket) highlights the risk of transmission of other families than EAI in the country. Although the Beijing family prevalence is still low, its presence mainly in the northern and central provinces, its association with drug resistance and its potential high involvement in recent transmission (clustering rate = 20% based on the combination of spoligotyping and 24 loci MIRU-VNTR) indicate that this family may change TB epidemiological pattern in Lao PDR. This underlines the need to continue and reinforce the effort to maintain an efficient TB control and surveillance system in order to prevent the emergence of highly transmissible and drug-resistant strains in Lao PDR, as observed in neighboring countries.

## Additional files

Additional file 1: **Figure S1.** Neighbor joining tree based on the MIRU-VNTR and spoligotyping data showing the genetic relationships of 202 *M. tuberculosis* isolates from Lao PDR (PDF). From left to right: i) Neighbor joining tree based on the 24-locus MIRU-VNTR and spoligotyping data for the 202 isolates built using the MIRU-VNTRplus analysis tool; ii) Number of repetitions of each VNTR according to the nomenclature by Supply et al. 2006); and iii) 43-spacer spoligotypes: black spots indicate the presence and white spot the absence of the 1–43 spacers (according to the numbering by Van Embden et al. 2000 [23]). Yellow squares, Beijing clusters; orange squares, EAI clusters; dark pink, T clusters. (PDF 7484 kb)

Additional file 2: **Table S1.** Complete data (clinical, epidemiological, demographic and genetic data) for the 222 *Mycobacterium tuberculosis* isolates included in this study. (XLSX 71 kb)

## Data Availability

The dataset supporting the conclusions of this article is included within the article and its additional files (Additional file 1: Figure S1 and Additional file 2: Table S1).

## Abbreviations

BCG: Bacillus Calmette Guérin
CAS: Central Asian Strain
CILM: Center of Infectiology Lao-Christophe Mérieux
CXR: Chest x-ray
DNA: Deoxyribonucleic acid
DOTS: Directly Observed Treatment Short course
EAI: East African-Indian
H: Haarlem
INH: Isoniazid
LAM: Latin American Mediterranean
MDR-TB: Multidrug resistant tuberculosis
MIRU-VNTR: Mycobacterial Interspersed Repetitive Unit-Variable Number Tandem Repeat
NJ: Neighbor joining
NRL: National Tuberculosis Reference Laboratory
NTCP: National Tuberculosis Control Program
RIF: Rifampicin
SITs: Spoligotype International Types
TB: Tuberculosis
WHO: World health organization
XDR-TB: Extensively drug-resistant tuberculosis

## References

[1] WHO | Global tuberculosis report 2016. WHO. http://www.who.int/tb/publications/global_report/en/. Accessed 1 Feb 2017.

[2] Coker RJ, Hunter BM, Rudge JW, Liverani M, Hanvoravongchai P (2011). Emerging infectious diseases in Southeast Asia: regional challenges to control. Lancet.

[3] Law I, Sylavanh P, Bounmala S, Nzabintwali F, Paboriboune P, Iem V (2015). The first national tuberculosis prevalence survey of Lao PDR (2010–2011). Tropical Med Int Health.

[4] Iem V, Somphavong S, Buisson Y, Steenkeste N, Breysse F, Chomarat M (2013). Resistance of *Mycobacterium tuberculosis* to antibiotics in Lao PDR: first multicentric study conducted in 3 hospitals. BMC Infect Dis.

[5] Chen YY, Chang JR, Huang WF, Hsu CH, Cheng HY, Sun JR, Kuo SC, Su IJ, Lin MS, Chen W, Dou HY (2017). Genetic diversity of the *Mycobacterium tuberculosis* East African-Indian family in three tropical Asian countries. J Microbiol Immunol Infect.

[6] Ismail F, Couvin D, Farakhin I, Rahman ZA, Rastogi N, Suraiya S (2014). Study of *Mycobacterium tuberculosis* complex genotypic diversity in Malaysia reveals a predominance of ancestral East-African-Indian lineage with a Malaysia-specific signature. PLoS One.

[7] Yu Q, Su Y, Lu B, Ma Y, Zhao X, Yang X (2013). Genetic diversity of *Mycobacterium tuberculosis* isolates from Inner Mongolia, China. PLoS One.

[8] Phyu S, Stavrum R, Lwin T, Svendsen ØS, Ti T, Grewal HMS (2009). Predominance of *Mycobacterium tuberculosis* EAI and Beijing lineages in Yangon, Myanmar. J Clin Microbiol.

[9] Zhang J, Heng S, Le Moullec S, Refregier G, Gicquel B, Sola C (2011). A first assessment of the genetic diversity of *Mycobacterium tuberculosis* complex in Cambodia. BMC Infect Dis.

[10] Nguyen VAT, Choisy M, Nguyen DH, Tran THT, Pham KLT, Dinh PTT (2012). High prevalence of Beijing and EAI4-VNM genotypes among *M. tuberculosis* isolates in northern Vietnam: sampling effect, rural and urban disparities. PLoS One.

[11] Nguyen VAT, Bañuls A-L, Tran THT, Pham KLT, Nguyen TS, Nguyen HV (2016). *Mycobacterium tuberculosis* lineages and anti-tuberculosis drug resistance in reference hospitals across Viet Nam. BMC Microbiol.

[12] Priorities for tuberculosis bacteriology services in low-income countries. The Union. http://www.theunion.org/what-we-do/publications/technical/priorities-for-tuberculosis-bacteriology-services-in-low-income-countries. Accessed 3 Feb 2017.

[13] MTBDRPLUS.pdf. http://tbevidence.org/documents/rescentre/sop/MTBDRPLUS.pdf. Accessed 23 July 2018.

[14] Filliol I, Driscoll JR, van Soolingen D, Kreiswirth BN, Kremer K, Valétudie G (2003). Snapshot of moving and expanding clones of *Mycobacterium tuberculosis* and their global distribution assessed by spoligotyping in an international study. J Clin Microbiol.

[15] Kamerbeek J, Schouls L, Kolk A, van Agterveld M, van Soolingen D, Kuijper S, Bunschoten A, Molhuizen H, Shaw R, Goyal M, van Embden J (1997). Simultaneous detection and strain differentiation of *Mycobacterium tuberculosis* for diagnosis and epidemiology. J Clin Microbiol.

[16] Demay C, Liens B, Burguière T, Hill V, Couvin D, Millet J (2012). SITVITWEB – a publicly available international multimarker database for studying *Mycobacterium tuberculosis* genetic diversity and molecular epidemiology. Infect Genet Evol.

[17] Vitola I, Driscoll J, Kreiswirth B, Kurepina N, Bennett P (2006). K. Identifying *Mycobacterium tuberculosis* complex strain families using spoligotypes. Infect Genet Evol.

[18] Supply P, Allix C, Lesjean S, Cardoso-Oelemann M, Rüsch-Gerdes S, Willery E (2006). Proposal for standardization of optimized mycobacterial interspersed repetitive unit-variable-number tandem repeat typing of *Mycobacterium tuberculosis*. J Clin Microbiol.

[19] Gauthier M, Bidault F, Mosnier A, Bablishvili N, Tukvadze N, Somphavong S (2015). High-throughput mycobacterial interspersed repetitive-unit–variable-number tandem-repeat genotyping for *Mycobacterium tuberculosis* epidemiological studies. J Clin Microbiol.

[20] Allix-Béguec C, Harmsen D, Weniger T, Supply P, Niemann S (2008). Evaluation and strategy for use of MIRU-VNTRplus, a multifunctional database for online analysis of genotyping data and phylogenetic identification of *Mycobacterium tuberculosis* complex isolates. J Clin Microbiol.

[21] van Deutekom H, Hoijng SP, de Haas PEW, Langendam MW, Horsman A, van Soolingen D (2004). Clustered tuberculosis cases: do they represent recent transmission and can they be detected earlier?. Am J Respir Crit Care Med.

[22] Shamputa IC, Rigouts L, Eyongeta LA, Aila NAE, van Deun A, Salim AH (2004). Genotypic and phenotypic heterogeneity among *Mycobacterium tuberculosis* isolates from pulmonary tuberculosis patients. J Clin Microbiol.

[23] van Embden JDA, van Gorkom T, Kremer K, Jansen R, van der Zeijst BAM, Schouls LM. Genetic Variation and Evolutionary Origin of the Direct Repeat Locus of Mycobacterium tuberculosis Complex Bacteria. J Bacteriol. 2000;182:2393–401. 10.1128/jb.182.9.2393-2401.2000 PMC111299 10762237

[24] Dong H, Liu Z, Lv B, Zhang Y, Liu J, Zhao X (2010). Spoligotypes of *Mycobacterium tuberculosis* from different provinces of China. J Clin Microbiol.

[25] Ali A, Hasan Z, Jafri S, Inayat R, Hasan R (2014). *Mycobacterium tuberculosis* central Asian strain (CAS) lineage strains in Pakistan reveal lower diversity of MIRU loci than other strains. Int J Mycobacteriol.

[26] Gutierrez MC, Ahmed N, Willery E, Narayanan S, Hasnain SE, Chauhan DS (2006). Predominance of ancestral lineages of *Mycobacterium tuberculosis* in India. Emerg Infect Dis.

[27] UNFPA Lao People’s Democratic Republic | Results of population and housing census 2015 (English version). http://lao.unfpa.org/publications/results-population-and-housing-census-2015-english-version. Accessed 22 Jan 2018.

[28] Yang C, Shen X, Peng Y, Lan R, Zhao Y, Long B (2015). Transmission of *Mycobacterium tuberculosis* in China: a population-based molecular epidemiologic study. Clin Infect Dis.

[29] Wang J, Liu Y, Zhang C-L, Ji B-Y, Zhang L-Z, Shao Y-Z (2011). Genotypes and characteristics of clustering and drug susceptibility of *Mycobacterium tuberculosis* isolates collected in Heilongjiang Province, China. J Clin Microbiol.

[30] Iwamoto T, Grandjean L, Arikawa K, Nakanishi N, Caviedes L, Coronel J (2012). Genetic diversity and transmission characteristics of Beijing family strains of *Mycobacterium tuberculosis* in Peru. PLoS One.

[31] Niemann S, Diel R, Khechinashvili G, Gegia M, Mdivani N, Tang Y-W (2010). *Mycobacterium tuberculosis* Beijing lineage favors the spread of multidrug-resistant tuberculosis in the republic of Georgia. J Clin Microbiol.

[32] Pang Y, Zhou Y, Zhao B, Liu G, Jiang G, Xia H (2012). Spoligotyping and drug resistance analysis of *Mycobacterium tuberculosis* Strains from National Survey in China. PLoS One.

