# Supplementary figures and images for: First insights into the genetic characteristics and drug resistance of Mycobacterium tuberculosis population collected during the first national tuberculosis prevalence survey of Lao PDR (2010–2011)
Source: BMC Infect Dis. 2019 Oct 15;19:851. doi: 10.1186/s12879-019-4435-z (PMC6794770; doi:10.1186/s12879-019-4435-z)

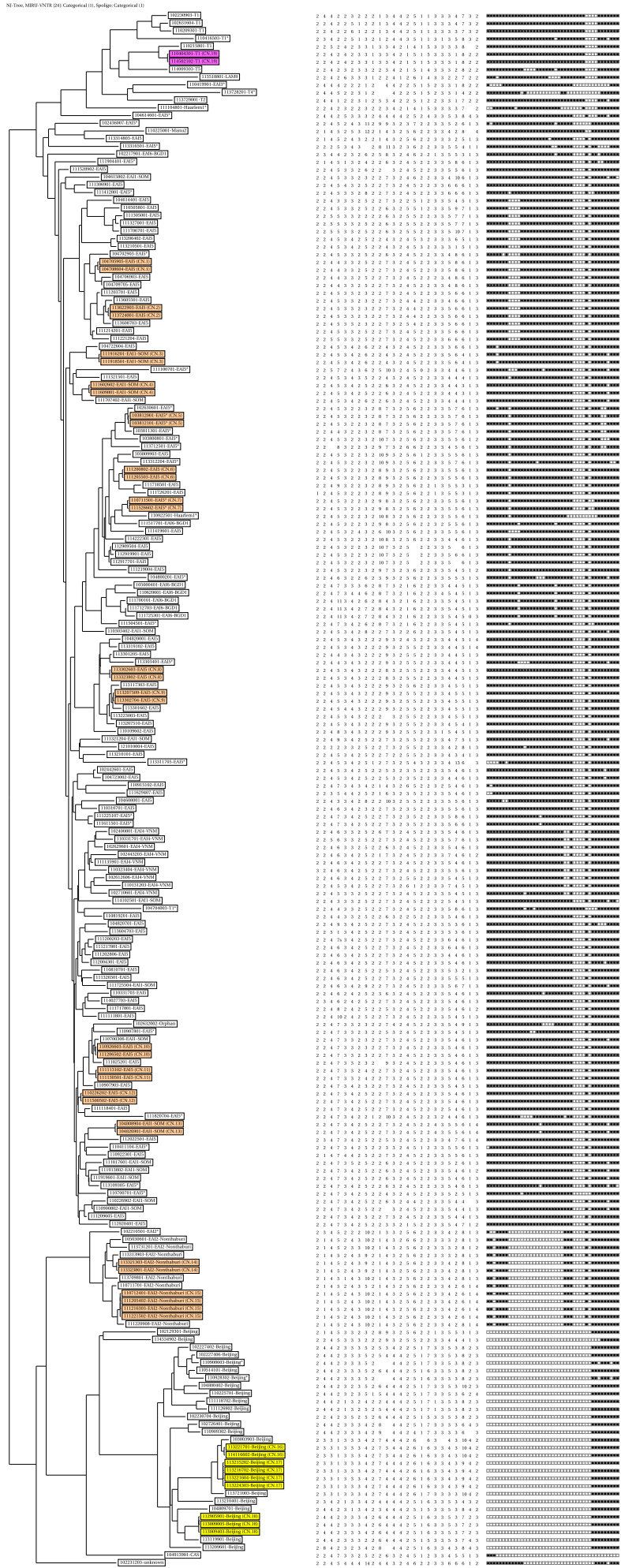

Supplement: Supplementary file 1 — Figure S1. Neighbor joining tree based on the MIRU-VNTR and spoligotyping data showing the genetic relationships of 202 M. tuberculosis isolates from Lao PDR (PDF). From left to right: i) Neighbor joining tree based on the 24-locus MIRU-VNTR and spoligotyping data for the 202 isolates built using the MIRU-VNTRplus analysis tool; ii) Number of repetitions of each VNTR according to the nomenclature by Supply et al. 2006); and iii) 43-spacer spoligotypes: black spots indicate the presence and white spot the absence of the 1–43 spacers (according to the numbering by Van Embden et al. 2000 [23]). Yellow squares, Beijing clusters; orange squares, EAI clusters; dark pink, T clusters. (PDF 7484 kb) [file 12879_2019_4435_MOESM1_ESM.pdf]
